# Abdominal Wall Endometriosis: A Case Report

**DOI:** 10.7759/cureus.4061

**Published:** 2019-02-13

**Authors:** Christian Saliba, Habib Jaafoury, Mohamed El Hajj, Gregory Nicolas, Houssein Haidar Ahmad

**Affiliations:** 1 General Surgery, Lebanese American University-Medical Center, Beirut, LBN; 2 General Surgery, Saint George Hospital/ Lebanese University, Beirut, LBN; 3 Obstetrics and Gynecology, Saher General Hospital / Lebanese University, Beirut, LBN; 4 General Surgery, Saint George Hospital/ Lebanese University, Beirut, LBN

**Keywords:** endometriosis, endometrioma, cesarean section

## Abstract

Abdominal wall endometriosis is a rare condition that occurs after a cesarean section or pelvic surgery and it has an incidence of 0.03%-1.5% in women with previous cesarean delivery. The predominant clinical picture is cyclic pain. We report two cases of abdominal wall endometriosis. The first was a 36-year-old female patient who presented for recurrent cyclic abdominal pain and was found to have endometriosis near the cesarean scar. The second was a 40-year-old female who had the same clinical presentation and was found to have endometriosis away from the scar. These cases highlight the need to have a high index of suspicion when treating women with recurrent cyclic abdominal pain.

## Introduction

Endometriosis was first described in 1860 [1]. It is defined as the presence or growth of endometrial tissue outside of the uterine cavity [2]. It occurs in 5%-10% of all women. Extra-pelvic endometriosis is rare, accounting for <12% of reported cases [2]. The various sites for extra-pelvic endometriosis are the bladder, kidney, bowel, omentum, lymph nodes, lungs, pleura, extremities, umbilicus, hernia sacs, and abdominal wall [3]. Medical management (nonsteroidal anti-inflammatory drugs (NSAIDs), oral contraceptives, and gonadotropin-releasing hormone agonists and aromatase inhibitors) has been the first line of treatment [4]. However, when symptoms become more and more refractory and start affecting the quality of life, a surgical approach is recommended for definitive management and diagnosis.

A rare spread of endometriosis is the abdominal wall endometrioma, which is commonly formed in a surgical scar resulting from a caesarian section delivery procedure. This category of endometriosis most commonly afflicts the cutaneous and subcutaneous tissue at the caesarean scar level; an intramuscular spread is a rare finding. Treatment modalities include surgical excision of the lesion and/or hormonal therapies. Wide surgical excision is still the treatment of choice in the literature [5]. Here in our case report, we present our experience with two rare findings of rectus muscle endometrioma to highlight the importance of surgical therapy in this subcategory of the disease.

## Case presentation

Case 1

**Figure 1:**
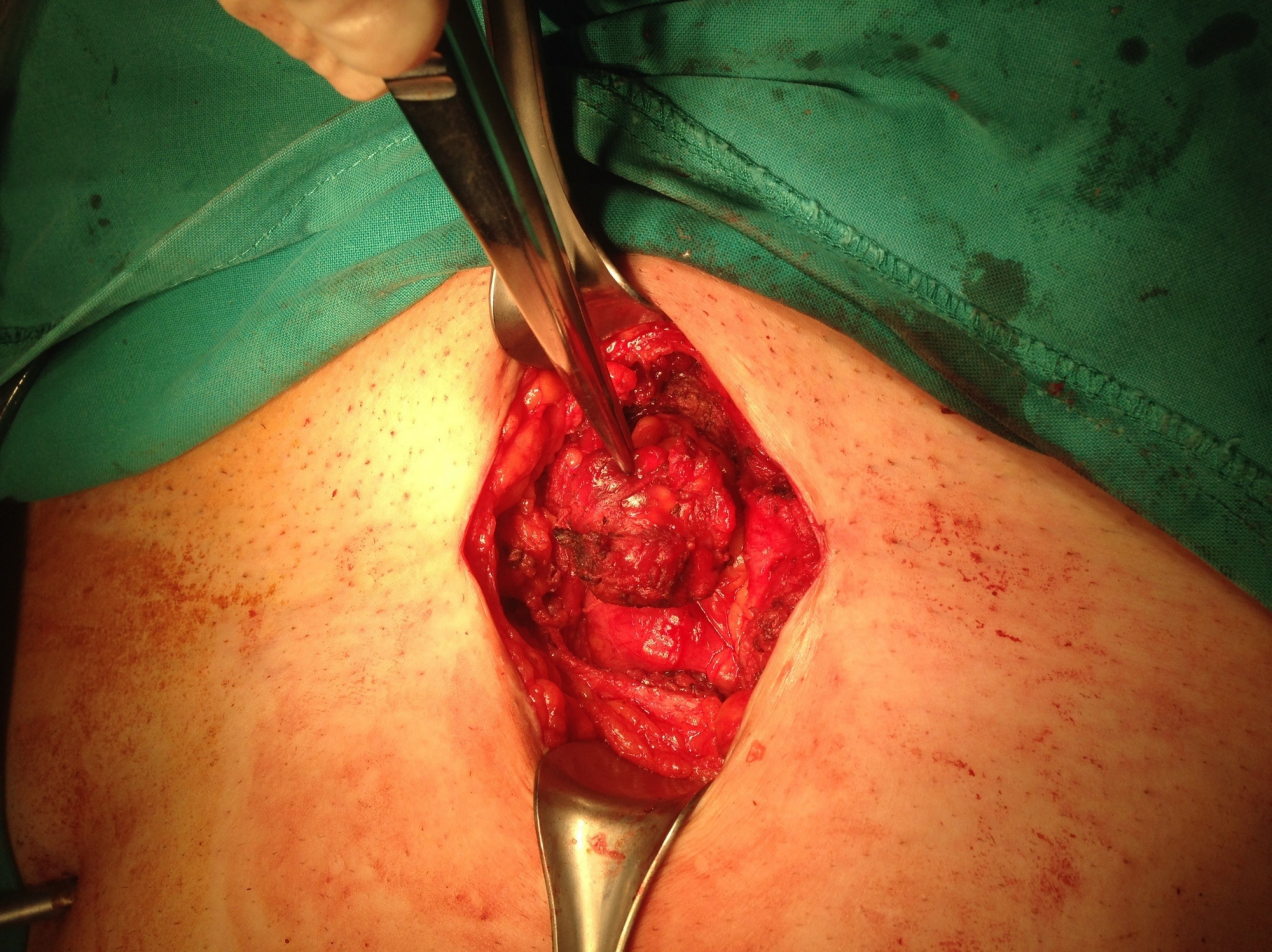
Intraoperative image showing the endometrioma in the subcutaneous tissue of the abdominal wall.

**Figure 2:**
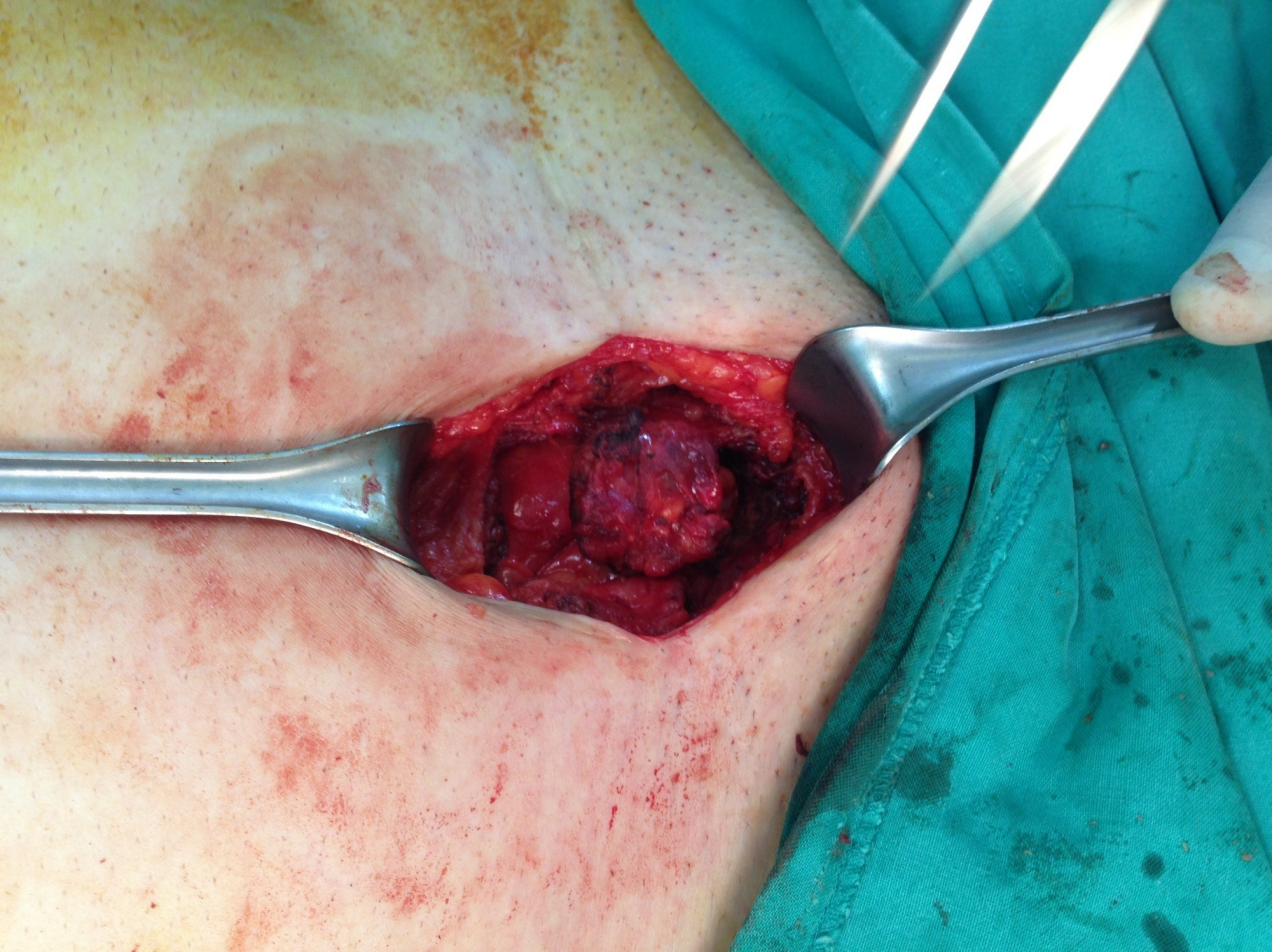
Intraoperative image showing the endometrioma in the subcutaneous tissue of the abdominal wall.

**Figure 3:**
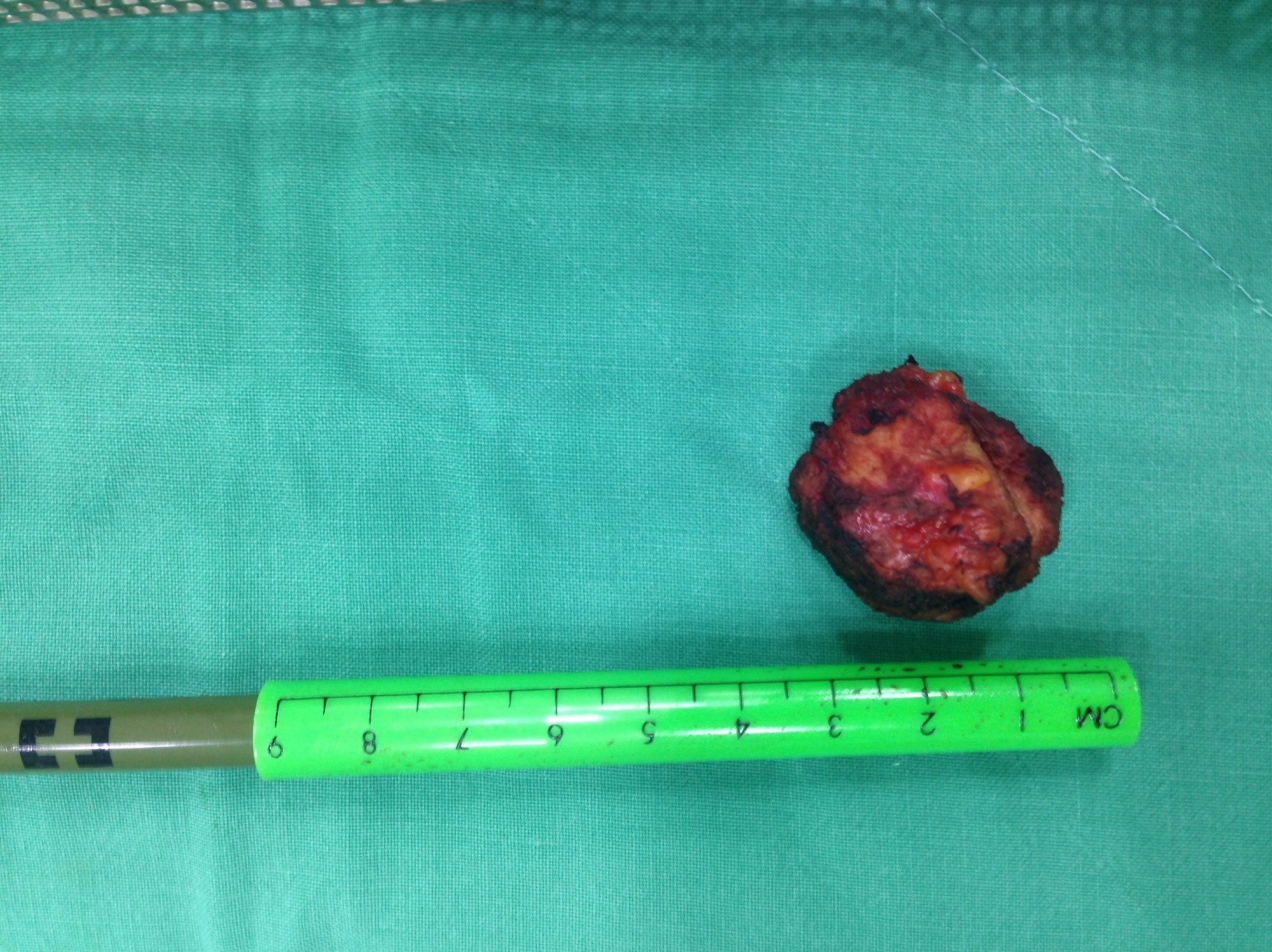
Image showing the resected endometrioma next to a graded ruler.

A 36-year-old female patient presented for recurrent abdominal pain and her past medical history was negative except for a cesarean delivery several years prior to her presentation. The pain was localized to the lower abdomen, crampy, cyclic, and worsened over the last few months. This pain was partially relieved by taking some analgesics. Hormonal therapy was afterwards tried for a month by taking progestins. The patient was no longer able to function properly in her daily tasks due to the pain. A physical exam revealed a slightly tender, non-mobile firm mass near the cesarean scar. A probable diagnosis of abdominal wall endometriosis was made. A computed tomography (CT) scan of the abdomen and pelvis with intravenous contrast revealed evidence of a homogeneous mass at the anterior abdominal wall just at the previous cesarean section showing slight enhancement. Under general anesthesia, surgical exploration (Figures 1, 2) revealed a 3×3 cm mass at the right lower rectus wall, and en bloc excision of the mass was performed (Figure 3). A pathologic examination showed pieces of benign thick fibrous tissue with multiple endometrial glands and stroma, diagnostic for endometriosis. The patient was seen at a regular interval, up until two months of follow-up and she was symptom free.

Case 2

**Figure 4:**
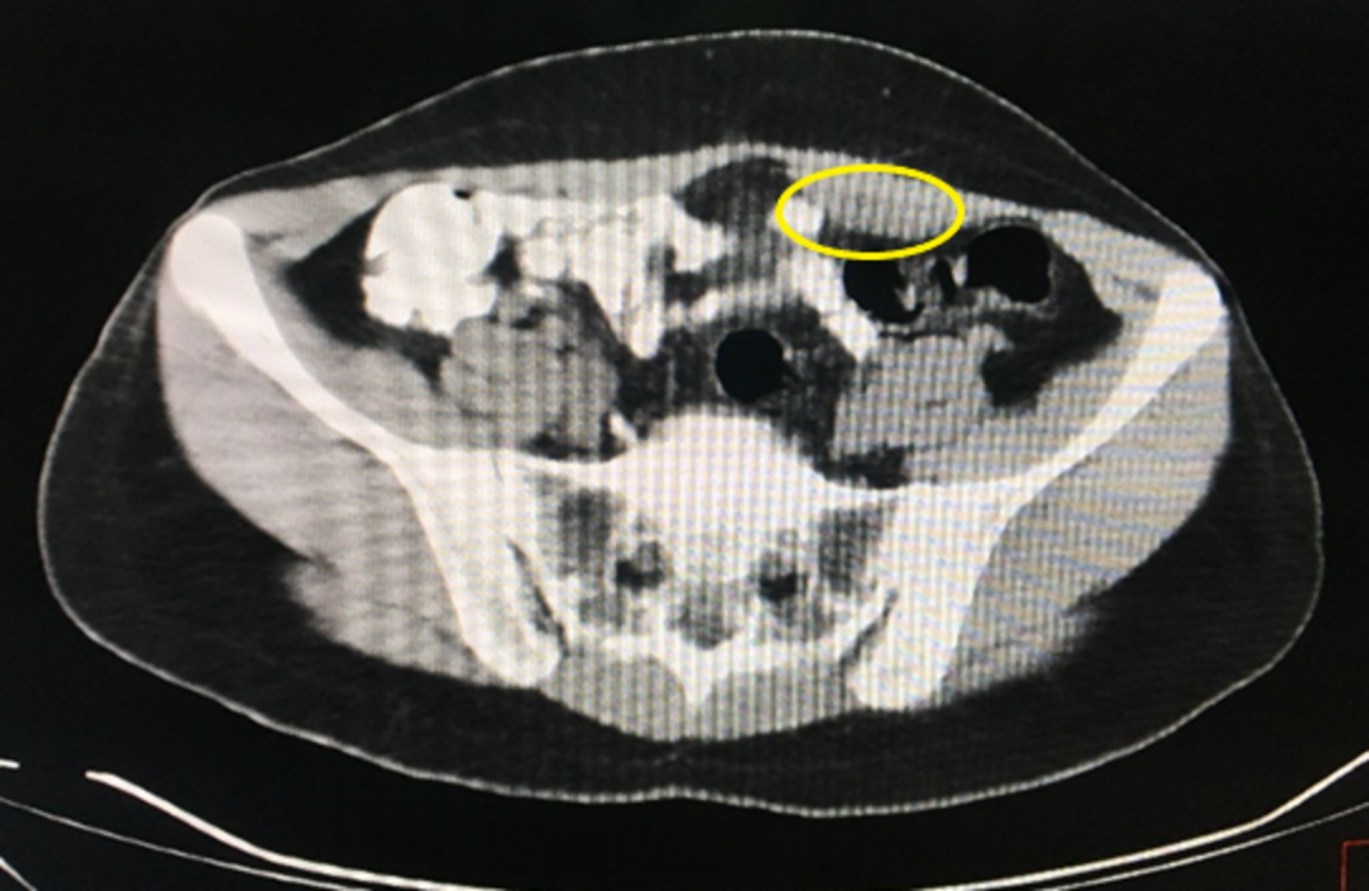
Computed tomography scan showing the endometrioma (yellow circle) contained in the rectus muscle, marked by a hypodensity lesion.

A 40-year-old female patient presented with the same clinical presentation of cyclic pain and her past history was negative except for a cesarean delivery several years ago. She noted that the pain was more at the left rectus muscle (away from the cesarean scar). She consulted her obstetrician at first who opted for medical treatment with analgesics and hormonal therapy (oral contraceptives and gonatropin-releasing hormone agonist), which were ineffective for her pain. A diagnosis of endometriosis was confirmed by an ultrasound and CT scan that showed a mid-rectus lesion (Figure 4). She underwent excision of the mass and mesh reinforcement was performed. Pathology confirmed the presence of endometrial stroma. The patient was disease-free and seen regularly up until a two-month interval.

## Discussion

Endometriosis is defined as the presence of ectopic endometrial tissue outside the uterine cavity [2]. It is classified as internal (within the uterine muscles) or external (in the pelvic organs and other parts of the body) according to the involvement of the uterine muscle layer. The most common pelvic organs affected are the ovaries, cul-de-sac, and fallopian tubes [6]. Extrapelvic locations include the gastrointestinal tract, pulmonary system, urinary system, abdominal wall, skin, and even the central nervous system [6]. The precise etiology of endometriosis remains controversial, and many theories have been proposed, including direct transplantation, coelomic metaplasia, cellular immunity, vascular and lymphatic metastasis, implantation and retrograde menstruation [7].

Abdominal wall endometriosis, often referred to as scar endometrioma, is a rare form of extragenital endometriosis [8]. The incidence of scar endometriosis following hysterotomy is 1.08%-2% whereas the incidence after cesarean section is 0.03%-0.4% [9]. The patient’s history and physical examination is very important and is usually highly suggestive in the diagnosis of scar endometriosis, especially in the presence of the classic presentation of a palpable mass, cyclic pain, and a previous incision due to cesarean delivery or gynecologic procedure [10]. Surgical scar endometriosis has a wide differential diagnosis such as a suture granuloma, inguinal or incisional hernia, lipoma or sebaceous cyst, etc. For that reason, the diagnosis should be confirmed by pathology [6].

Abdominal endometriosis can be detected by CT scan, ultrasound, and magnetic resonance imaging. On Doppler ultrasound, the masses appear as solid hypoechoic lesions in the abdominal wall containing vessels. There is no specific finding on CT or MRI except for a solid enhancing mass in the abdominal wall, and it is more useful to detect the extent of the disease preoperatively [6].

The treatment of choice is wide local excision of the lesion with negative margins and treatment may sometimes require mesh placement. Medical therapy including NSAIDs, oral contraceptives, gonadotropin-releasing hormone analogues and aromatase inhibitors are used and show improvements in symptoms with no change in the lesion size [6].

## Conclusions

Abdominal wall endometriosis is a very rare condition but it should always be kept in mind when treating females who present with recurrent cyclic abdominal pain. Definitive management and cure is provided by surgery with minimal consequences.
